# The Impact of COVID-19 on the Profile of Hospital-Acquired Infections in Adult Intensive Care Units

**DOI:** 10.3390/antibiotics10101146

**Published:** 2021-09-23

**Authors:** Aleksa Despotovic, Branko Milosevic, Andja Cirkovic, Ankica Vujovic, Ksenija Cucanic, Teodora Cucanic, Goran Stevanovic

**Affiliations:** 1 Faculty of Medicine, University of Belgrade, 11000 Belgrade, Serbia; b.milosevic@yahoo.com (B.M.); ankica.vujovic88@gmail.com (A.V.); goran_drste@yahoo.com (G.S.); 2 Teaching Hospital for Infectious and Tropical Diseases, University Clinical Center of Serbia, 11000 Belgrade, Serbia; xeniac995@gmail.com (K.C.); teodorac995@gmail.com (T.C.); 3 Department of Medical Statistics, Faculty of Medicine, University of Belgrade, 11000 Belgrade, Serbia; andja.cirkovic@mfub.bg.ac.rs

**Keywords:** hospital-acquired infections, COVID-19, intensive care unit, adults, antimicrobial resistance, empiric therapy, carbapenem resistance, trend analysis, surveillance, Serbia

## Abstract

Hospital-acquired infections (HAIs) are a global public health concern. As the COVID-19 pandemic continues, its contribution to mortality and antimicrobial resistance (AMR) grows, particularly in intensive care units (ICUs). A two-year retrospective study from April 2019–April 2021 was conducted in an adult ICU at the Hospital for Infectious and Tropical Diseases, Belgrade, Serbia to assess causative agents of HAIs and AMR rates, with the COVID-19 pandemic ensuing halfway through the study. Resistance rates >80% were observed for the majority of tested antimicrobials. In COVID-19 patients, *Acinetobacter* spp. was the dominant cause of HAIs and more frequently isolated than in non-COVID-19 patients. (67 vs. 18, *p* = 0.001). Also, resistance was higher for imipenem (56.8% vs. 24.5%, *p* < 0.001), meropenem (61.1% vs. 24.3%, *p* < 0.001) and ciprofloxacin (59.5% vs. 36.9%, *p* = 0.04). AMR rates were aggregated with findings from our previous study to identify resistance trends and establish empiric treatment recommendations. The increased presence of *Acinetobacter* spp. and a positive trend in *Klebsiella* spp. resistance to fluoroquinolones (R^2^ = 0.980, *p* = 0.01) and carbapenems (R^2^ = 0.963, *p* = 0.02) could have contributed to alarming resistance rates across bloodstream infections (BSIs), pneumonia (PN), and urinary tract infections (UTIs). Exceptions were vancomycin (16.0%) and linezolid (2.6%) in BSIs; tigecycline (14.3%) and colistin (0%) in PNs; and colistin (12.0%) and linezolid (0%) in UTIs. COVID-19 has changed the landscape of HAIs in our ICUs. Approval of new drugs and rigorous surveillance is urgently needed.

## 1. Introduction

The burden of hospital-acquired infections (HAIs) on healthcare systems and patients is substantial and constantly growing. Recent surveys show that up to 4.5 million individuals suffer from at least one HAI in acute care hospitals across Europe every year [1,2,3]. Appropriate infection prevention and control has been particularly challenging since the start of the COVID-19 pandemic, during which studies are showing an increase in HAI occurrence [4,5,6]. Prior to the pandemic, evidence suggested that up to 55% of HAIs could be prevented through effective preventative practices [7]. During the pandemic, however, the sudden and enormous demands on healthcare systems has inevitably reduced the quality of infection control worldwide. Furthermore, the use of large amounts of antibiotics and immunosuppressive therapy in COVID-19 patients has only accentuated the problem of antimicrobial resistance (AMR) and the incidence of multidrug-resistant (MDR) organisms [8,9].

AMR is of particular concern in intensive care units (ICUs), where choices for empiric therapy are limited. Pathogens such as carbapenem-resistant *Enterobacteriaceae* (CRE) and carbapenem-resistant *Acinetobacter baumannii (CRA)* have become hard to treat as carbapenems, the mainstay of both empiric and targeted therapy, are increasingly not fit for use [10]. This has become even more important during the pandemic, as outbreaks of both CRE and CRA are increasingly reported worldwide [11,12,13,14], affecting patient outcomes that are already compromised due to COVID-19. Because of these challenges, rigorous local surveillance is critical, as it enables continuous revisions of empiric therapy guidelines and improvement in antimicrobial stewardship, both for initiation of therapy and its de-escalation [15,16].

In Serbia, AMR for HAIs has been evaluated through national point prevalence surveys and isolated reports from various hospitals [17,18,19], but no studies detailing the profile of HAIs and AMR have been published since the beginning of the pandemic in our country. In our previous two-year study conducted before the pandemic [20], we identified risk factors for both acquisition and mortality in patients with HAIs in adult ICUs and discovered high resistance rates to most tested antibiotics. In this two-year study, spanning from April 2019–April 2021, we further investigated the profile of pathogens and their susceptibility to antibiotics currently used for treatment. We compared AMR rates of pathogens isolated before the pandemic (1 April 2019–31 March 2020) and during the pandemic (1 April 2020–31 March 2021), with the goal of assessing potential differences in causative agents and their antimicrobial susceptibility rates (ASTs). The primary aim, however, was to establish multi-year trends and assess which drugs can still be used for empiric and/or targeted therapy for most common HAIs in ICUs based on local resistance profiles, with respect to the impact of COVID-19.

## 2. Results

A total of 611 patients were admitted to the ICU of the Teaching Hospital for Infectious and Tropical Diseases in Belgrade, Serbia between April 2019 and April 2021. Before the pandemic, the majority of patients treated at our ICU were those suffering from severe forms of central nervous system (CNS), respiratory, systemic and other infections. Since the beginning of the pandemic, however, our hospital and our ICU serve as one of the leading facilities for treatment of patients with severe COVID-19 in the country.

During the study period, a total of 114 patients suffered from at least one HAI, with their clinical and demographic characteristics shown in Table 1. 35.1% of patients were female, and the median length of stay (LOS) was 24 days (IQR 23). The principal diagnosis for which patients were treated was COVID-19 infection (*n* = 73, 64.0%) and comprised the majority of patients (93.6%) admitted to our ICU during the second year of our study. 58 patients (50.9%) were directly admitted to our ICU from our ambulatory center, whereas 56 (49.1%) of patients were transferred from other departments of our hospital and other departments from the University Clinical Center of Serbia. Notable comorbidities included cardiovascular disease (*n* = 83; 73.5%), diabetes (*n* = 34; 30.1%) and chronic lung disease (*n* = 14; 12.4%). All patients received antibiotics 48h before or after admission, most common being cephalosporins (*n* = 63, 55.3%), vancomycin (*n* = 42, 36.8%) and carbapenems (*n* = 38, 33.3%).

More than 1 HAI was identified in 53 (46.5%) of patients and a third of all HAIs were polymicrobial. Therefore, a total of 163 HAI episodes with 226 isolates were confirmed. The most commonly diagnosed HAIs were bloodstream infections (BSI; *n* = 69, 42.3%), pneumonia (PN; *n* = 63, 38.7%), and urinary tract infections (UTI; *n* = 15, 9.2%). Other HAIs were of the skin and soft-tissue (SST; *n* = 10, 6.1%), central nervous system (CNS; *n* = 4, 2.5%), and 1 episode of otitis externa (EENT) and vaginitis (REPR) each, respectively. 89% of both PNs and UTIs were classified as device-associated (intubation/ventilation-associated pneumonia, IAP/VAP; and catheter-associated urinary tract infection; CAUTI, respectively). Bacterial pathogens isolated as causative agents of HAIs during our study period are shown in Table 2. *Acinetobacter* spp. was the most frequently identified pathogen (*n* = 85, 37.6%), primarily isolated from the respiratory tract (*n* = 57, 72.2%). Other isolates included *Enterococcus* spp., coagulase-negative *Staphylococcus* (CoNS), *Klebsiella* spp., and *Pseudomonas aeruginosa.* 176 pathogens (79.3%) were classified as MDR. A concomitant *Clostridium difficile* infection was found in 17 patients (12.5%).

Antimicrobial susceptibility testing (AST) revealed alarming resistance rates to almost all tested antibiotics (Table 3). The highest rates were observed against fluoroquinolones (levofloxacin, 97.4%; ciprofloxacin, 96.4%; moxifloxacin, 94.4%) and cephalosporins (cefotaxime 96.3%, cephalexin 95.8%, ceftriaxone 95.7%, ceftazidime 90.0%), with overall rates exceeding 90% for both groups. Resistance to trimethoprim-sulfamethoxazole (TMP-SMX) was 88.8%, whereas resistance of >80% was also seen for both aminoglycosides (91.6% and 80.4% for gentamicin and amikacin, respectively). With the exception of amoxicillin-clavulanic acid (75.0%), resistance rates exceeded > 80% in all other penicillins (methicillin/oxacillin, 92.3%; piperacillin-tazobactam, 87.8%; ampicillin/amoxicillin, 80.4%). Resistance to carbapenems was also high (85.4% for meropenem and 81.3% for imipenem), while resistance to ertapenem, though on a much smaller sample size (*n* = 23) was lower (47.8%). Resistance to vancomycin (30.8%) and tigecycline (28.6%) is lower compared to most drugs, while colistin (7.1%) and linezolid (2.7%) showed the lowest resistance rates.

In addition to overall resistance rates, we investigated any potential difference in pathogens causing HAIs in COVID-19 patients compared to non-COVID-19 patients in the pre-pandemic year (Table 4). An association between the diagnosis of COVID-19 and increased resistance to all carbapenems (imipenem, 56.8% vs. 24.5%, *p* < 0.001; meropenem, 61.1% vs. 24.3%, *p* < 0.001; and ertapenem, 26.1% vs. 21.7%, *p* = 0.03) has been observed. Ciprofloxacin was the only other antibiotic in which a statistically significant association was identified (59.5% vs. 36.9%, *p* = 0.04). We also see an association between increased numbers of PNs and the diagnosis of COVID-19 (48 vs. 15, *p* = 0.008), but also an inverse association between UTIs and the diagnosis of COVID-19 (3 vs. 12, *p* < 0.001). The analysis of pathogen distribution shows an association between *Acinetobacter* spp. as a causative agent of HAIs and a diagnosis of COVID-19 (67 vs. 18, *p* = 0.001) and an inverse association of *Proteus mirabilis* and COVID-19 (2 vs. 6, *p* = 0.02).

**Figure 1:**
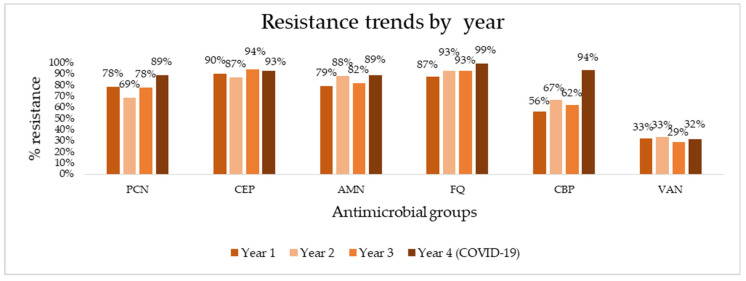
Antimicrobial resistance trends for major antibiotics over four years of surveillance. PCN, penicillins; CEP, cephalosporins; AMN, aminoglycosides; FQ, fluoroquinolones; CBP, carbapenems; VAN, vancomycin.

To determine the appropriate options for empiric and targeted treatment of HAIs, we aggregated overall resistance rates of major antimicrobial groups (penicillins, cephalosporins, aminoglycosides, fluoroquinolones, carbapenems, and vancomycin) from this study and our previous study and assessed trends in resistance over four years of follow-up (Figure 1). Absolute numbers of resistance rates can be found in Supplementary Table S1. The aggregation of data shows consistently high resistance rates, reaching 90% in the last year of follow-up to virtually all antibiotic groups—99% for fluoroquinolones, 94% for carbapenems, 93% for cephalosporins, and 89% for penicillins. Vancomycin is the exception, exhibiting much lower resistance rates (32% in the last year). A sharp spike in resistance is seen for carbapenems, rising to from 62% to 94% in the last year. Using linear regression and trend analysis (Table 5), we identified that there is a borderline statistically significant positive trend for pathogen resistance to fluoroquinolones (R^2^ = 0.904, *p* = 0.05) and a positive, but not statistically significant trend for carbapenems (R^2^ = 0.704, *p* = 0.16). 

**Figure 2:**
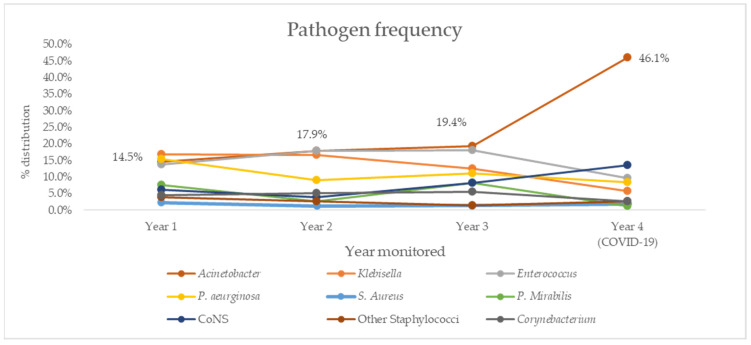
Distribution of most commonly isolated causative agents of HAIs in our ICU over four years of follow-up. CoNS, coagulase-negative *Staphylococcus*.

Trends for overall resistance rates were further investigated by looking at pathogen distribution in the same period (Figure 2) and trend analysis (Table 6). Notable results include a negative, but not statistically significant trend for *Klebsella* spp. (R^2^ = 0.863, *p* = 0.08) and positive, but not statistically significant trend for *Acinetobacter* spp. (R^2^ = 0.721, *p* = 0.15), which was the most common cause of both PNs and BSIs in our study.

**Figure 3:**
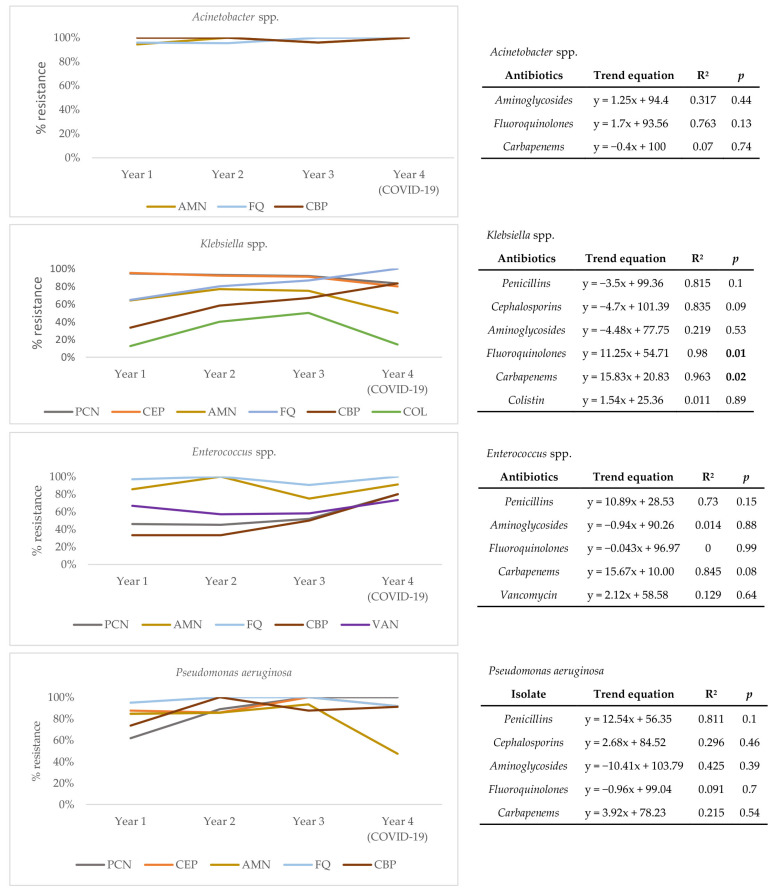
Trends of antimicrobial resistance for *Acinetobacter* spp., *Klebsiella* spp., *Enterococcus* spp., and *Pseudomonas aeruginosa* isolated over four years of follow-up. AMN, aminoglycosides; FQ, fluoroquinolones; CBP, carbapenems; PCN, penicillins; COL, colistin; VAN, vancomycin; R^2^—coefficient of determination; statistically significant values (*p* < 0.05) marked in bold.

To understand the drivers of resistance from the perspective of causative agents, a trend analysis for each of the four most frequently isolated pathogens over four years of follow-up (*Acinetobacter* spp., *Klebsiella* spp., *Enterococcus* spp., *and Pseudomonas aeruginosa*) was conducted (Figure 3). Results show that the resistance profile of *Acinetobacter* spp. has not significantly changed over time. However, a statistically significant positive trend for resistance to fluoroquinolones (R^2^ = 0.980, *p* = 0.01) and carbapenems (R^2^ = 0.963, *p* = 0.02) is seen for *Klebsiella* spp., pointing to a change in its resistance profile. Though not statistically significant, a positive trend of resistance to carbapenems (R^2^ = 0.845, *p* = 0.08) is also seen in *Enterococcus* spp. Absolute numbers of pathogen distribution and resistance rates of individual pathogens can be found in the Supplementary Table S2.

The final analysis of our study aggregates AST results over the same four-year period and evaluates which drugs could still be used as empiric treatment for the three most commonly identified HAIs in our ICU-BSIs, PNs, and UTIs (Table 7).

For causative agents of BSIs we see significant resistance rates to all major antimicrobial groups—97.2% for cephalosporins, 94.8% for fluoroquinolones, 86.3% for penicillins, and 80.8% for carbapenems. Resistance to vancomycin (16.0%), tigecycline (13.9%), colistin (12.9%), and linezolid (2.6%) remains low. Similar results are seen in PNs, where only tigecycline (14.3%) and colistin (0%) exhibit low resistance rates. When it comes to UTIs, very high resistance rates are present for cephalosporins (93.3%), fluoroquinolones (90.2%), TMP-SMX (87.5%) and aminoglycosides (85.9%). Somewhat lower, but still high resistance rates are present for penicillins (66.2%), vancomycin (61.1%) and carbapenems (53.1%), with only colistin (12.0%) and linezolid (0%) exhibiting low resistance rates to pathogens responsible for this type of infection.

## 3. Discussion

Our study describes the profile of HAIs in our ICU over a two-year period and their change in profile during the COVID-19 pandemic. The resistance rates of HAIs to the majority of antibiotics that are currently approved for empiric, but also targeted therapy in our country are extremely high. Compared to all EU/EEA countries and international reports, our findings show much higher resistance rates, with only Romania and Greece reporting somewhat comparable results for certain antimicrobial groups [1,21]. Furthermore, our results are consistent with the Central Asian and European Surveillance of Antimicrobial Resistance (CAESAR) reports from our country, where resistance to pathogens such as *A. baumannii* exceeds 90% for all tested antibiotics [22]. The only drugs that exhibited fairly low overall resistance rates were linezolid and colistin. Linezolid resistance across Europe and worldwide remains low [23,24], but colistin-resistant HAIs in ICUs are becoming a concern [25,26]. Colistin plays a crucial role in treatment of MDR infections, and efforts toward determining colistin resistance on a larger scale are ongoing [27]. Studies in our country have indeed identified emerging colistin resistance with similar results [28].

The problem of such high AMR in Serbia stems, at least in part, from poor overall antimicrobial stewardship. Prescribing rates have significantly grown over the past decade are much higher than the European average [29]. Furthermore, the excessive and irrational use of antibiotics occurs through self-prescribing practices that are still possible in our country, but also poor adherence to national guidelines for rational antimicrobial use [30,31]. This is unfortunate, as studies from Serbian hospitals have shown that resistance rates can decline through adequate surveillance and changes in antibiotic prescribing [32]. Education plays a significant role as well, with recent studies showing a limited understanding regarding proper use of antibiotics in Serbian general population. [33] Our findings, although dealing with HAIs rather than community-acquired infections, reiterate the critical role of local surveillance in determining optimal therapeutic strategies [1,16]. 

When it comes to COVID-19 and its impact on HAIs in our study, we see a significant shift in the type of infection, causative agents, and resistance profiles. Though not unexpected, the rise of PNs, many of which caused by CRA, brings a new challenge when it comes to HAI management. Increased rates of HAIs in ICUs that treat COVID-19 patients are being reported in various countries and support our findings [4,34], likely as a result of the inability to fully comply with the standard practices of infection control during this unprecedented time [12,35,36]. Another risk for promoting resistance in patients hospitalized for COVID-19 is the use of immunosuppressive therapy, though studies are showing conflicting results regarding its contribution to resistance [37,38]. Serbia’s national guideline for COVID-19 treatment (provided in Supplementary Table S3) emphasizes the absolute avoidance of antibiotic use precisely because of the risk of rising AMR. Moreover, in-hospital use of antibiotics has known to be a driver of resistance in a time-dependent manner [39], pointing to a need for rational use of antimicrobials across all levels of care.

The trend analysis of antimicrobial resistance shows that the increased presence of *Acinetobacter* spp. (with stable resistance to fluoroquinolones and carbapenems over the years), coupled with the statistically significant positive trend of *Klebsiella* spp. resistance to the two antibiotic groups, could be responsible for the current situation in our ICU. Carbapenem-resistant *Klebsiella* infections in COVID-19 patients treated in ICUs have indeed been documented [13,14]. Knowing the mechanism by which resistance developed could be crucial in determining optimal infection control practices and new therapeutic options as the pandemic continues. The acquisition of resistance is known to be an important component of overall antimicrobial stewardship for HAIs in ICUs [40,41,42]. Coupled with investigating this, infection control efforts aimed at *Acinetobacter* spp. and *Enterobacteriaceae* should be rigorously implemented to help reduce their occurrence in our ICU [43,44].

This brings us to the final topic of our study—choice of empiric therapy. In the case of BSIs, we see that currently used penicillins, cephalosporins, aminoglycosides, fluoroquinolones, and carbapenems are not fit for use as empiric, or even targeted therapy. Vancomycin is still a viable antibiotic for empiric treatment of BSIs according to our results, but is at risk of reaching higher rates of resistance already appearing in other countries [45,46].

For PNs, virtually all drugs available in our country, also listed in major international guidelines, exhibited very high resistance rates, thus limiting their use as empiric therapy [47,48]. Colistin and tigecycline remain the only antibiotics in our setting, though their use in such capacity is not substantiated by high-quality evidence [48,49,50]. In fact, tigecycline is not recommended if *Acinetobacter* spp. are causative agents of PN, which was predominantly the case in our study [48,51].

When it comes to UTIs, we see a similar situation in that high resistance rates are seen for most tested antibiotics. Because most UTIs in our ICU are CAUTIs, the use of amoxicillin, aminoglycosides, third-generation cephalosporins, and ciprofloxacin as empiric therapy, all of them recommended in European guidelines, is not viable [52]. In addition, resistance rates of 61.1% for vancomycin makes a difficult case for its use as empiric therapy. Unless urosepsis is suspected, deferring therapy until AST reveals good candidates for targeted treatment may be a wiser approach [53], as Linezolid and tigecycline seem to be only viable options for empiric CAUTI treatment in our setting. 

Decreasing the time from obtaining samples for analysis to getting results could help in facilitating earlier initiation of treatment for HAIs in general. The development of “rapid antimicrobial susceptibility testing” (RAST) provides AST results within hours [54], but a number of other methods are being developed and used with equal reduction in time to results [55,56]. Such practices must become the gold standard in our laboratories, as earlier initiation of effective therapy significantly improves patient outcomes [57].

The need for new drugs, especially as the COVID-19 pandemic continues, is perhaps the most important step our country needs to take in order to successful manage HAIs, both for COVID-19 patients and across other ICUs. In recent years, several drugs have been approved for treatment of MDR pathogens and different types of HAIs in the European Union (EU) and the United States (US) [58,59,60], both as standalone drugs and combination therapies, whereas a number of drugs are being investigated through clinical trials [61,62]. Aztreonam, considered an “old” drug, has also shown to be a useful agent in treating MDR infections as an adjunct to some of the recently approved antibiotics [63,64]. With our findings, the case has been made for urgent approval of new drugs for HAI treatment in Serbia.

There are several limitations of our study that need to be pointed out, starting with those related to the retrospective study design. First, we were limited in our capacity to look at the relationship between the choice of empiric therapy, its potential change to targeted therapy and the overall effects of HAIs on patient outcomes. Second, it also limited our ability to look at the interaction between antimicrobial use that extends beyond window of 48h before and after admission, given the established relationship of prolonged antimicrobial use and development of resistance [65]. And third, the extent to which immunosuppressive drugs (such as corticosteroids and/or tocilizumab) used in COVID-19 treatment played a role in the development of AMR [12].

Additionally, our results are from only one type of ICU, that of treating exclusively infectious diseases and currently dealing with COVID-19 patients. By including other types of ICUs such as surgical, cardiovascular, and pediatric/neonatal, thereby increasing the sample size as well, would help understand the potential differences in pathogen profiles and resistance rates. These findings could improve national recommendations for empiric therapy and put a stronger emphasis on both new drug approval and better diagnostics. 

Finally, the inconsistencies in AST seen above are a consequence of implementing the European Committee on Antimicrobial susceptibility testing (EUCAST) recommendations that vary between pathogens [54], and in some instances, our inability to adhere to the recommendations to their full extent. As a result, we were not able to establish rates of extensive-drug resistant and pan-drug resistant (XDR/PDR) pathogens in our ICU [66]. To perform such classification, comprehensive panels of testing are required, including drugs that are not currently available for use in our country.

## 4. Materials and Methods

### 4.1. Study Design

This two-year retrospective study included all patients that were admitted and discharged between April 2019 and April 2021 from the 16-bed adult ICU of the 150-bed Teaching Hospital for Infectious and Tropical Diseases, University Clinical Center of Serbia. Since the start of the COVID-19 pandemic, which coincided with the beginning of the second year of follow-up in our study, the ICU of our hospital has been tasked with primarily caring for severe COVID-19 patients requiring intensive care. During the two-year period, health care records from a total of 673 patients over 18 years old were evaluated (as our hospital treats only adult patients). Criteria for exclusion were a length of stay < 48 h (*n* = 58) and incomplete patient record data (*n* = 4). Patients in whom a diagnosis of HAI as a result of stay in our ICU was not made (*n* = 497) were excluded from the final analysis.

Patients in whom one or more distinct HAI episodes were diagnosed and for which AST results were available were included in the study. Comparisons between causative agents of HAIs and their resistance rates were made in COVID-19 patients, who comprised the majority of patients during the second year of our study (1 April 2020–31 March 2021), and patients without a COVID-19 diagnosis, the majority being admitted during the pre-pandemic year (1 April 2019–31 March 2020). The obtained results from this study were then aggregated with data from our previous study to derive four-year trends of resistance across different antimicrobial groups and overall resistance of pathogens responsible for causing the most frequently identified HAIs—BSIs, PNs, and UTIs. In addition, pathogen frequencies and trends of antimicrobial resistance for four of the most frequently isolated pathogens over the same period (*Acinetobacter* spp., *Klebsiella* spp., *Enterococcus* spp., and *Pseudomonas aeruginosa*) to major antibiotic groups were investigated.

### 4.2. Definitions and Data Collection

Each episode of ICU-acquired HAI was defined and categorized using the European Center for Disease Control (eCDC) criteria, occurring ≥48 hours after admission, with onset from day 3 onwards and day 1 being the date of admission [67]. These criteria allowed us to exclude patients in whom HAIs developed before admission to our ICU, such as cases in which HAIs developed in facilities where they were previously hospitalized. As a result, HAI from our ICU types included bloodstream infections (BSI), pneumonia (PN), urinary tract infections (UTI), central nervous system infection (CNS), eye, ear, nose or mouth infection (EENT), skin and soft tissue infections (SST), and reproductive tract infections (REPR) [68]. Assessment of multidrug resistant (MDR) pathogens was conducted using standardized susceptibility criteria—resistance to at least one antibiotic from at least three groups of antimicrobial drugs [66]. Both AST and pathogen identification at our hospital is conducted using Vitek2 ^®^bioMerieux, guided by the EUCAST breakpoints and recommendations, with intermediately resistant results being classified as resistant [69]. The antibiotic groups that have been tested included: penicillins (methicillin-oxacillin, MET-OXA; ampicillin/amoxicillin, AMP/AMX; amoxicillin-clavulanic acid, AMX-CL; piperacillin-tazobactam, PIP-TZ), cephalosporins (cephalexin, cefotaxime, ceftriaxone, ceftazidime), aminoglycosides (gentamicin, amikacin), fluoroquinolones (ciprofloxacin, levofloxacin, moxifloxacin), carbapenems (imipenem, meropenem, ertapenem), glycopeptides (vancomycin, teicoplanin), trimethoprim-sulfamethoxazole (TMP-SMX), linezolid, tigecycline, and colistin.

Data collected from patient records included basic demographic and clinical characteristics (age, biological sex, date of admission and discharge to determine LOS, type of admission, primary diagnosis, comorbidities, antimicrobial use 48h prior to and after admission). Additionally, dates of invasive device placement and removal, including urinary catheters, central venous catheters, respiratory tubes, and mechanical ventilation was collected to determine if HAIs were device-associated, in line with eCDC criteria [67]. Dates of HAI sampling and source of acquisition were collected, together with AST results. 

### 4.3. Data Analysis

The Statistical Package for Social Sciences (SPSS) software version 23 (IBM Corp. Released 2015. IBM SPSS Statistics for Windows, Version 23.0. Armonk, NY, USA: IBM Corp.) was used for analysis of patient data. Mean and standard deviation were used to describe variables that exhibited normal distribution, whereas median and interquartile range (IQR) were used to describe variables that did not exhibit normal distribution. Numbers and percentages were used to describe the distribution of pathogens responsible for HAIs, as well as antimicrobial resistance rates, both overall and stratified across most common HAI types.

Chi-square test was used to determine the association between a COVID-19 diagnosis and the following variables: resistance rates to individual antibiotics, infection types (PN, BSI, UTI), and individual pathogens (*Acinetobacter* spp., *Klebsiella* spp., coagulase-negative *Staphylococcus*, *Pseudomonas aeruginosa*, *Enterococcus* spp., *Proteus mirabilis*). Using linear regression, we investigated trends of antimicrobial resistance rates and pathogen distribution across four years of follow-up. For antimicrobial resistance rates, trend analysis was firstly conducted by combining all pathogens responsible for HAIs across six antimicrobial groups (penicillins, cephalosporins, aminoglycosides, fluoroquinolones, carbapenems, and vancomycin). This was followed by a separate analysis for the most frequently isolated agents over four years of follow-up (*Acinetobacter* spp., *Klebsiella* spp., *Enterococcus* spp., and *Pseudomonas aeruginosa*) for applicable antimicrobial groups. For all statistical tests used in the study, *p* values < 0.05 were considered statistically significant.

All data that were included in the analysis were previously anonymized. The manuscript was prepared in accordance with the STROBE statement, attached in Supplementary Table S4.

## 5. Conclusions

As a result of managing COVID-19 patients, our study showed a significant shift in the landscape of HAIs in our ICU compared to previous years. *Acinetobacter* spp. and *Klebsiella* spp. are pathogens that are driving resistance to carbapenems and fluoroquinolones, leaving limited options for empiric and targeted therapy. Immediate approval of new drugs for treatment of hospital-acquired BSIs, PNs, and UTIs is needed. Implementation of faster microbiological diagnostics is necessary to ensure early initiation of optimal treatment for every patient. Surveillance needs to be continued and expanded both in terms of antibiotics subjected to testing and other ICUs to revise local treatment guidelines as the pandemic continues.

## Figures and Tables

**Table 1:** Clinical characteristics of 114 patients who suffered from at least one HAI in our study from April 2019–April 2021.

Patient Characteristics	*n* = 114 (%)
Age	66.2 (±13.6 years)
Length of Stay	24 (IQR 23)
Sex (Female)	40 (35.1%)
Primary Diagnosis	
COVID-19 Infection	73 (64.0%)
CNS infection	18 (15.8%)
Other respiratory infections	6 (5.3%)
Admission to ICU	
Directly admitted	58 (50.9%)
Transferred from other departments	56 (49.1%)
Comorbidities	
Cardiovascular Disease	83 (73.5%)
Diabetes	34 (30.1%)
Chronic lung disease	14 (12.4%)
Kidney insufficiency	7 (6.2%)
Hypothyroidism	6 (5.3%)
Invasive device use	
Urinary catheter	110 (96.5%)
Central venous line	60 (54.1%)
Intubation	101 (88.6%)
Mechanical ventilation	99 (86.8%)
Antibiotic use 48 before and after admission	114 (100%)
Penicillins	11 (9.6%)
Cephalosporins	63 (55.3%)
Aminoglycosides	3 (2.6%)
Fluoroquinolones	20 (17.5%)
Carbapenems	38 (33.3%)
Vancomycin	42 (36.8%)
>1 HAI	53 (46.5%)
Polymicrobial infection	55 (33.7%)

*n*: number of patients; CNS: central nervous system; ICU: intensive care unit; HAI: hospital-acquired infection; IQR: interquartile range.

**Table 2:** Causative agents of HAIs that underwent AST from 1 April 2019–31 March 2021.

Isolates	*n* = 226 (%)	BSI (*n* = 98)	PN (*n* = 79)	UTI (*n* = 19)
*Acinetobacter* spp.	85 (37.6)	26 (26.5)	57 (72.2)	2 (10.5)
*Enterococcus* spp.	28 (12.4)	11 (11.2)	2 (2.5)	8 (42.1)
Coagulase-negative *Staphylococcus*	27 (11.9)	26 (26.5)		
*Pseudomonas aeruginosa*	21 (9.3)	3 (3.1)	11 (13.9)	2 (10.5)
*Klebsiella* spp.	21 (9.3)	7 (7.1)	5 (6.3)	5 (26.3)
*Proteus mirabilis*	8 (3.5)	1 (1.0)		1 (5.3)
*Escherichia coli*	5 (2.2)	1 (1.0)		1 (5.3)
*Providencia* spp.	5 (2.2)	2 (2.0)		
Other Staphylococcal species	5 (2.2)	5 (5.1)		
*Staphylococcus aureus*	4 (1.8)	2 (2.0)	2 (2.5)	
Diphtheroids	4 (1.8)	3 (3.1)	1 (1.3)	
*Stenotrophomonas maltophilla*	4 (1.8)	3 (3.1)	1 (1.3)	
*Corynebacterium* spp.	3 (1.3)	3 (3.1)		
*Achromobacter xylooxidans*	3 (1.3)	3 (3.1)		
*Prevotella* spp.	1 (0.4)	1 (1.0)		
*Kocuria kristinae*	1 (0.4)	1 (1.0)		

*n*, number of isolates; BSI, bloodstream infection; PN, pneumonia; UTI, urinary tract infection.

**Table 3:** Antimicrobial susceptibility testing results from April 2019–April 2021.

Antibiotics (*n*)	% Resistance	Antibiotics (*n*)	% Resistance
Penicillins		Cephalosporins	
MET/OXA (39)	92.3%	Cephalexin (24)	95.8%
AMP/AMX (56)	80.4%	Cefotaxime (27)	96.3%
AMX-CL (40)	75.0%	Ceftriaxone (46)	95.7%
PIP-TZ (41)	87.8%	Ceftazidime (60)	90.0%
Total (176)	83.5%	Total (157)	93.6%
Fluoroquinolones		Carbapenems	
Ciprofloxacin (168)	96.4%	Imipenem (139)	81.3%
Levofloxacin (156)	97.4%	Meropenem (144)	85.4%
Moxifloxacin (36)	94.4%	Ertapenem (23)	47.8%
Total (360)	96.7%	Total (330)	74.8%
Aminoglycosides		Glycopeptides	
Gentamicin (166)	91.6%	Vancomycin (65)	30.8%
Amikacin (143)	80.4%	Teicoplanin (11)	63.6%
Total (309)	86.5%	Total (76)	35.6%
Other Antibiotics		Other Antibiotics	
TMP-SMX (134)	88.8%	Colistin (127)	7.1%
Tigecycline (21)	28.6%	Linezolid (36)	2.7%

*n*, number of pathogens tested; MET, methicillin; OXA, oxacillin; AMP, ampicillin; AMX, amoxicillin; AMX-CL, amoxicillin-clavulanic acid; PIP-TZ, piperacillin-tazobactam; TMP-SMX, trimethoprim-sulfamethoxazole.

**Table 4:** Resistance rates, types of infection, and pathogen distribution in COVID-19 vs. non-COVID-19 patients.

Antibiotics (*n*)	COVID-19	Non-COVID-19	*p*
	(*n* = 73)	(*n* = 41)	
MET/OXA (39)	71.80%	20.50%	0.16
AMP/AMX (56)	39.30%	41.10%	1
AMX_CL (40)	32.50%	42.50%	0.71
PIP_TZ (41)	39.00%	48.80%	0.38
Cefalexin (24)	29.20%	66.70%	1
Cefotaxim (27)	22.20%	74.10%	1
Ceftriaxon (46)	34.80%	60.90%	0.54
Ceftazidime (60)	31.70%	58.30%	0.19
Gentamicin (166)	57.20%	34.30%	0.16
Amikacin (143)	53.80%	26.60%	0.38
Ciprofloxacin (168)	59.50%	36.90%	**0.04**
Levofloxacin (156)	62.20%	35.30%	0.14
Moxifloxacin (36)	47.20%	47.20%	0.49
Bactrim (134)	58.20%	30.60%	0.78
Imipenem (139)	56.80%	24.50%	**<0.001**
Meropenem (144)	61.10%	24.30%	**<0.001**
Ertapenem (23)	26.10%	21.70%	**0.03**
Tigycycline (21)	9.50%	19.00%	0.36
Linezolid (36)	0.00%	2.80%	1
Colistin (127)	3.10%	3.90%	0.11
Vancomycin (65)	20.00%	10.80%	1
**Type of Infection**	**COVID-19**	**Non-COVID-19**	***p***
	**(*n* = 73)**	**(*n* = 41)**	
PN	48	15	**0.008**
BSI	48	21	0.19
UTI	3	12	**<0.001**
Pathogens	**N = 147**	**N = 79**	
*Acinetobacter* spp.	67	18	**0.001**
*Enterococcus* spp.	15	13	0.21
CoNS	21	6	0.2
*Pseudomonas aeruginosa*	12	9	0.47
*Klebsiella* spp.	8	10	0.07
*Proteus mirabilis*	2	6	**0.02**

*n*, number of patients; N, number of isolated pathogens; MET, methicillin; OXA, oxacillin; AMP, ampicillin; AMX, amoxicillin; AMX-CL, amoxicillin-clavulanic acid; PIP-TZ, piperacillin-tazobactam; TMP-SMX, trimethoprim-sulfamethoxazole. PN, pneumonia; BSI, bloodstream infection; UTI, urinary tract infection; CoNS, coagulase-negative *Staphylococcus*; Statistically significant values (*p* < 0.05) in bold.

**Table 5:** Trend analysis for major antimicrobial groups over four years of follow-up.

Isolate	Trend Equation	R^2^	*p*
*Penicillins*	y = 4.13x + 68.10	0.416	0.35
*Cephalosporins*	y = 1.48x + 87.18	0.344	0.41
*Aminoglycosides*	y = 2.15x + 79.22	0.350	0.41
*Fluoroquinolones*	y = 3.54x + 84.14	0.904	0.05
*Carbapenems*	y = 10.75x + 42.79	0.700	0.16
*Vancomycin*	y = −0.65x + 33.31	0.223	0.53

R^2^—coefficient of determination.

**Table 6:** Trend analysis for most frequently isolated causes of HAI over four years of follow-up.

Isolate	Trend Equation	R^2^	*p*
*Acinetobacter* spp.	y = 9.63x + 0.40	0.721	0.15
*Enterococcus* spp.	y = −1.18x + 17.80	0.146	0.62
Coagulase-negative *Staphylococcus*	y = 2.70x + 1.20	0.692	0.17
*Pseudomonas aeruginosa*	y = −1.86x + 15.60	0.591	0.23
*Klebsiella* spp.	y = −3.72x + 22.25	0.863	0.07
*Proteus mirabilis*	y = −1.32x + 8.25	0.235	0.51
Other Staphylococcal species	y = −0.48x + 3.80	0,400	0.37
*Staphylococcus aureus*	y = −0.11x + 2.00	0.093	0.69
*Corynebacterium* spp.	y = −0.55x + 5.85	0.292	0.46

R^2^—coefficient of determination; statistically significant values (*p* < 0.05) marked in bold.

**Table 7:** Four-year antimicrobial resistance rates of BSIs, PNs, and UTIs in our ICU.

Antibiotics	BSI (*n* = 158)	PN (*n* = 123)	UTI (*n* = 108)
**Penicillins**			
MET/OXA	92.9% (52/56)	100% (5/5)	N/A
AMP/AMX	84.8% (28/33)	87.5% (14/16)	68.5% (37/54)
AMX-CL	80.0% (12/15)	80% (12/15)	59.3% (32/54)
PIP-TZ	77.8% (21/27)	90.9% (30/33)	70.5% (31/44)
Total	86.3% (113/131)	88.4% (61/69)	66.2% (100/151)
**Cephalosporins**			
Cephalexin	100% (7/7)	N/A	100% (12/12)
Cefotaxime	100% (8/8)	N/A	100% (12/12)
Ceftriaxone	97.4% (38/39)	93.9% (31/33)	88.0% (44/50)
Ceftazidime	94.1% (16/17)	76.9% (10/13)	100% (16/16)
Total	97.2% (69/71)	89.1% (41/46)	93.3% (84/90)
**Aminoglycosides**			
Gentamicin	89.9% (98/109)	93.4% (85/91)	88.9% (64/72)
Amikacin	74.1% (60/81)	83.2% (79/95)	82.1% (46/56)
Total	83.2% (158/190)	88.2% (164/186)	85.9% (110/128)
**Fluoroquinolones**			
Ciprofloxacin	95.0% (96/101)	96.8% (92/95)	93.6% (73/78)
Levofloxacin	94.2% (98/104)	92.2% (95/103)	86.7% (65/75)
Moxifloxacin	96.4% (27/28)	N/A	N/A
Total	94.8% (221/233)	94.4% (187/198)	90.2% (138/153)
**Carbapenems**			
Imipenem	83.3% (35/42)	95.2% (60/63)	68.8% (11/16)
Meropenem	84.4% (38/45)	97.2% (69/71)	90.0% (9/10)
Total *	80.8% (97/120)	91.3% (158/173)	53.1% (43/81)
**Other Antibiotics**			
TMP-SMX	N/A	93.8% (75/80)	87.5% (35/40)
Tigecycline	13.9% (5/36)	14.3% (1/7)	27.8% (5/18)
Linezolid	2.6% (1/39)	N/A	0% (0/21)
Colistin	12.9% (8/62)	0% (0/98)	12.0% (3/25)
Vancomycin	16.0% (12/75)	20% (1/5)	61.1% (22/36)

*n*, number of pathogens isolated; BSI, bloodstream infection; PN, pneumonia; UTI, urinary tract infection; MET, methicillin; OXA, oxacillin; AMP, ampicillin; AMX, amoxicillin; AMX-CL, amoxicillin-clavulanic acid; PIP-TAZ, piperacillin-tazobactam; TMP-SMX, trimethoprim-sulfamethoxazole. N/A, no results available; * In years 1 and 2 of follow-up (during our previous study), carbapenem resistance was noted as resistance to either imipenem or meropenem.

## Data Availability

The majority of the data supporting the results of this study are located in the Supplementary Materials of this manuscript. Any additional information can be available on request to the corresponding author.

## Supplementary Materials

The following are available online at https://www.mdpi.com/article/10.3390/antibiotics10101146/s1, Table S1: Resistance rates of each antibiotic included in trend analysis over a four-year period; Table S2: Absolute numbers of pathogens distribution and resistance rates of individual pathogens; Table S3: Serbian National COVID-19 Treatment Guidelines; Table S4: STROBE Checklist for Cohort studies.
