# Supplementary material for: The Impact of COVID-19 on the Profile of Hospital-Acquired Infections in Adult Intensive Care Units
Source: Antibiotics (Basel). 2021 Sep 23;10(10):1146. doi: 10.3390/antibiotics10101146 (PMC8532680; doi:10.3390/antibiotics10101146)
Supplement: Supplementary file 1 [file antibiotics-10-01146-s001.zip › antibiotics-1375672 revised Supplementary Tables.pdf]

Supplementary Table S1

Absolute numbers of resistance rates for individual antibiotics and overall resistance for each major antimicrobial group for each year of follow-up

**Year 1**	% resistance	R (n)	Total tested
**Penicillins**			
Met/OXA	93.3%	14	15
AMP/AMX	82.6%	38	46
AMX-CL	71.1%	27	38
PIP-TZ	75.4%	43	57
**Total**	**78.2%**	**122**	**156**
**Cephalosporins**			
Ceftriaxone	**90.1%**	**64**	**71**
**Aminoglycosides**			
Gentamicin	84.4%	76	90
Amikacin	72.9%	51	70
**Total**	**79.4%**	**127**	**160**
**Fluoroquinolones**			
Ciprofloxacin	90.7%	78	86
Levofloxacin	84.6%	88	104
**Total**	**87.4%**	**166**	**190**
**Carbapenems***** (Imipenem/Meropenem)	**56.3%**	**49**	**87**
**Vancomycin**	**32.5%**	**13**	**40**

R, number of results indicating resistance; n, number of cases; MET, methicillin; OXA, oxacillin; AMP, ampicillin; AMX, amoxicillin; AMX-CL, amoxicillin-clavulanic acid; PIP-TAZ, piperacillin-tazobactam; TMP-SMX, trimethoprim-sulfamethoxazole. N/A, no results available;

*****In Years 1 & 2, carbapenem resistance was identified as resistance to either Imipenem or Meropenem**

Year 2	% resistance	R (n)	Total tested
**Penicillins**			
Met/OXA	90.0%	9	10
AMP/AMX	63.0%	17	27
AMX-CL	52.0%	13	25
PIP-TZ	85.7%	18	21
**Total**	**68.7%**	**57**	**83**
**Cephalosporins**			
Ceftriaxone	**86.7%**	**26**	**30**
**Aminoglycosides**			
Gentamicin	90.2%	46	51
Amikacin	86.4%	38	44
**Total**	**88.4%**	**84**	**95**
**Fluoroquinolones**			
Ciprofloxacin	94.4%	51	54
Levofloxacin	89.3%	50	56
**Total**	**92.7%**	**101**	**109**
**Carbapenems** (Imipenem/Meropenem)	**66.7%**	**36**	**54**
**Vancomycin**	**33.3%**	**8**	**24**

R, number of results indicating resistance; n, number of cases; MET, methicillin; OXA, oxacillin; AMP, ampicillin; AMX, amoxicillin; AMX-CL, amoxicillin-clavulanic acid; PIP-TAZ, piperacillin-tazobactam; TMP-SMX, trimethoprim-sulfamethoxazole. N/A, no results available;

*****In Years 1 & 2, carbapenem resistance was identified as resistance to either Imipenem or Meropenem**

**Year 3**	% resistance	R (n)	Total tested
**Penicillins**			
Met/OXA	80.0%	8	10
AMP/AMX	79.3%	23	29
AMX-CL	69.6%	16	23
PIP-TZ	83.3%	20	24
Total	77.9%	67	86
**Cephalosporins**			
Cefalexin	94.1%	16	17
Cefotaxime	95.2%	20	21
Ceftriaxone	93.1%	27	29
Ceftazidime	94.4%	34	36
**Total**	**94.2%**	**97**	**103**
**Aminoglycosides**			
Gentamicin	86.7%	52	60
Amikacin	75.6%	34	45
**Total**	**81.9%**	**86**	**105**
**Fluoroquinolones**			
Ciprofloxacin	91.9%	57	62
Levofloxacin	94.3%	50	53
Moxifloxacin	89.5%	17	19
Norfloxacin	100.0%	5	5
**Total**	**92.8%**	**129**	**139**
**Carbapenems**			
Imipenem	58.3%	28	48
Meropenem	65.9%	29	44
**Total**	**62.0%**	**57**	**92**
Vancomycin	29.2%	7	24

R, number of results indicating resistance; n, number of cases; MET, methicillin; OXA, oxacillin; AMP, ampicillin; AMX, amoxicillin; AMX-CL, amoxicillin-clavulanic acid; PIP-TAZ, piperacillin-tazobactam; TMP-SMX, trimethoprim-sulfamethoxazole. N/A, no results available;

Year 4 Total	% resistance	R (n)	Total tested
**Penicillins**			
Met/OXA	96.6%	28	29
AMP/AMX	81.5%	22	27
AMX-CL	82.4%	14	17
PIP-TZ	94.1%	16	17
**Total**	**88.9%**	**80**	**90**
**Cephalosporins**			
Cefalexin	100.0%	7	7
Cefotaxime	100.0%	6	6
Ceftriaxone	100.0%	17	17
Ceftazidime	83.3%	20	24
**Total**	**92.6%**	**50**	**54**
**Aminoglycosides**			
Gentamicin	94.3%	100	106
Amikacin	82.7%	81	98
**Total**	**88.7%**	**181**	**204**
**Fluoroquinolones**			
Ciprofloxacin	99.1%	105	106
Levofloxacin	99.0%	102	103
Moxifloxacin	100.0%	17	17
Norfloxacin	100.0%	1	1
**Total**	**99.1%**	**225**	**227**
**Carbapenems**			
Imipenem	93.4%	85	91
Meropenem	94.0%	94	100
**Total**	**93.7%**	**179**	**191**
Vancomycin	31.7%	13	41

R, number of results indicating resistance; n, number of cases; MET, methicillin; OXA, oxacillin; AMP, ampicillin; AMX, amoxicillin; AMX-CL, amoxicillin-clavulanic acid; PIP-TAZ, piperacillin-tazobactam; TMP-SMX, trimethoprim-sulfamethoxazole. N/A, no results available;

Supplementary Table S2 Absolute numbers of pathogen distribution and resistance rates of most frequently identified pathogens over four years of follow-up

Acinetobacter spp.	AMN			FQ			CBP		
	n	R	%	n	R	%	n	R	T
Year 1	18	17	94%	24	23	96%	19	19	100%
Year 2	16	16	100%	22	21	95%	14	14	100%
Year 3	24	23	96%	24	24	100%	25	24	96%
Year 4 (COVID-19)	106	106	100%	106	106	100%	115	115	100%

n, number of tested antibiotics; R, number of resistant pathogens; AMN, aminoglycosides; FQ, fluoroquinolones; CBP, carbapenems;

Klebsiella spp.	PCN			CEP			AMN			FQ			CBP			COL		
	n	R	%	n	R	%	n	R	%	n	R	%	n	R	%	n	r	%
Year 1	37	35	95%	21	20	95%	28	18	64%	34	22	65%	21	7	33%	8	1	13%
Year 2	14	13	93%	13	12	92%	13	10	77%	20	16	80%	12	7	58%	10	4	40%
Year 3	12	11	92%	22	20	91%	12	9	75%	15	13	87%	18	12	67%	4	2	50%
Year 4 (COVID-19)	12	10	83%	10	8	80%	12	6	50%	12	12	100%	24	20	83%	7	1	14%

n, number of tested antibiotics; R, number of resistant pathogens; PCN, penicillins; CEP, cephalosporins; AMN, aminoglycosides; FQ, fluoroquinolones; CBP, carbapenems; COL, colistin;

Enterococcus spp.	PCN			AMN			FQ			CBP			VAN		
	n	R	%	n	R	%	n	R	%	n	R	%	n	r	%
Year 1	37	17	46%	14	12	86%	33	32	97%	6	2	33%	18	12	67%
Year 2	31	14	45%	25	25	100%	21	21	100%	12	4	33%	14	8	57%
Year 3	27	14	52%	12	9	75%	21	19	90%	8	4	50%	12	7	58%
Year 4 (COVID-19)	25	20	80%	11	10	91%	6	6	100%	10	8	80%	15	11	73%

n, number of tested antibiotics; R, number of resistant pathogens; PCN, penicillins; AMN, aminoglycosides; FQ, fluoroquinolones; CBP, carbapenems; VAN, vancomycin

P. aeruginosa	PCN			CEP			AMN			FQ			CBP			COL		
	n	R	%	n	R	%	n	R	%	n	R	%	n	R	%	n	r	%
Year 1	21	13	62%	16	14	88%	39	33	85%	39	37	95%	19	14	74%	13	0	0%
Year 2	9	8	89%	7	6	86%	14	12	86%	14	14	100%	7	7	100%	5	0	0%
Year 3	8	8	100%	7	7	100%	15	14	93%	15	15	100%	16	14	88%	8	0	0%
Year 4 (COVID-19)	12	12	100%	12	11	92%	19	9	47%	24	22	92%	22	20	91%	12	0	0%

n, number of tested antibiotics; R, number of resistant pathogens; PCN, penicillins; CEP, cephalosporins; AMN, aminoglycosides; FQ, fluoroquinolones; CBP, carbapenems; COL, colistin;

Supplementary Table S3 Serbian National guidelines for COVID-19 Treatment, Version 11. (Translated from Serbian to English and the original Serbian Version)

**Protocol for the treatment of COVID-19 patients**<br>Version 11		
**Form of the disease**	**Institution**	**Treatment**
FORM 1<br>1. **Positive nasopharyngeal swab** (PCR for SARS-CoV-2, Ag test)<br>**2. Asymptomatic**<br>**3. Very mild clinical presentation**<br>4. **Patients without comorbidities** and with the mild form of the infection (hospitalized patients with sO_2>94% and **without signs of pneumonia on X-ray**)	**At-home treatment and home isolation** with monitoring and follow-up by **Covid clinic staff**	<u>**Vitamin therapy**</u>: alphacalcidol tablets (**Alpha D3**), 1x2 mcg, **vitamin C** 1x 1g (if there are no kidney issues)<br><u>**Do not routinely prescribe antibiotics due to the risk of serious adverse effects and the rising resistance to antimicrobial drugs)*</u><br><u>**Antiviral therapy**</u> (**to be introduced no later than the 5th day** of symptom onset): **favipiravir** tablets, 1,600 mg every 12 h on the first day, then 600 mg every 12 h for another 4 days (the drug is applied in patients with symptoms)<br><u>**Symptomatic treatment**</u>
FORM 2<br>**1. Positive nasopharyngeal swab** (PCR for SARS-CoV-2, Ag test)<br>**2. Mild clinical presentation**<br>**3. Patients without comorbidities** and with the mild form of the infection (hospitalized patients with sO_2>94% and **with signs of pneumonia on X-ray, but without signs of hypoxia at admission**)	1. **Covid centers**: general wards<br>2. **General hospitals**: isolation units<br>Increased monitoring measures<br>Checking patient temperature, sO_2 and respiration rate several times a day	<u>**Anticoagulant therapy:**</u> low-molecular-weight heparin<br><u>**Antiviral therapy**</u> (**to be introduced no later than the 5th day** of symptom onset): **favipiravir** tablets, 1,600 mg every 12 h on the first day, then 600 mg every 12 h for another 4 days<br><u>**Corticosteroid therapy**</u><br><u>**Vitamin therapy**</u>: alphacalcidol tablets (**Alpha D3**), 1x2 mcg, **vitamin C** 1x1 g<br><u>**Convalescent plasma:**</u> (within 2 weeks of symptom onset and after consultation with an infectologist and transfusiologist, according to the score)<br><u>*Application of antibiotics only in likely or proven bacterial infection*</u>
FORM 3<br>**1. Positive nasopharyngeal swab** (PCR for SARS-CoV-2, Ag test)<br>**2. Moderate clinical presentation**<br>**3. Severe hypoxia** with necessary oxygen therapy (sO_2<90%), fever, multiple opacities on chest X-ray (or	1. **Covid centers**: semi-intensive care units<br>2. **General hospitals**: isolation units with <u>**constant**</u> monitoring (≤1h) of intensive care specialist, anesthesiologist, infectologist or internal medicine specialist	<u>**Anticoagulant therapy:**</u> low-molecular-weight heparin<br><u>**Vitamin therapy**</u>: alphacalcidol tablets (**Alpha D3**), 1x2 mcg, **vitamin C** 1x1 g<br><u>**Oxygen therapy**</u>: nasal catheter or mask O_2 (10 – 15 l/minute

characteristic changes in the lungs visible on CT)<br>4. **Cytokine storm** (deterioration of general health status with ↑CRP, ↑fibrinogen, ↑D-dimer, ↑IL-6) (at least one parameter elevated)	3. **<u>In case of further deterioration</u> transportation to Covid intensive care**	**<u>Antiviral therapy</u> (if less than 5 days** have elapsed since symptom onset): **favipiravir** (as above) or **remdesivir** ampules 200 mg intravenously on the first day, then 100 mg IV/day for another 4 days<br><br>(**<u>Corticosteroid therapy</u>** and/or **tocilizumab** 8 mg/kg IV per dose. Two doses are administered (max 800 mg per dose)<br><br>**<u>Antibiotic therapy</u>**: in keeping with the guidelines for rational application of antibiotics<br><br>**<u>Convalescent plasma:</u>** (within 2 weeks of symptom onset and after consultation with an infectologist and transfusiologist, according to the score)
FORMS 4 and 5<br><br>1. **Positive nasopharyngeal swab**<br>2. **Very severe/severe clinical presentation**<br>3. **Onset or development of ARDS**<br>4. **Cytokine storm** (deterioration of general health status with ↑CRP, ↑fibrinogen, ↑D-dimer, ↑IL-6)	**Covid centers**: intensive care units	**<u>All available</u>** <u>measures/combinations of measures need to be applied</u><br><br>**Maintaining ↑ O_2 flow** as long as possible, **MV** in case of further deterioration<br><br>**Corticosteroid therapy:** methylprednisolone 1-2 mg/kg, 3-5 days, then gradual reducing of the dose (dose should be adjusted to body mass in order to reduce the risk of bleeding and other adverse effects)<br><br>**Tocilizumab:** 8 mg/kg IV per dose. Two doses (max 800 mg per dose)<br><br>**Immunoglobulins:** 10 – 20 g per day Ig over a period of 3 – 5 days

PROTOKOL ZA LEČENJE PACIJENATA SA KOVID-19 VERZIJA 11

FORMA BOLESTI	USTANOVA	TERAPIJA
OBLIK 1<br>1. **Pozitivan nazofaringealni bris** (PCR na SARS-KoV-2, Ag test)<br>2. **Asimptomatska**<br>3. **Vrlo blaga klinička slika**<br>4. **Bolesnici bez komorbiditeta** i sa blagim oblikom infekcije (hospitalizovani pacijenti sa sO_2 > 94% i **bez Rtg znakova pneumonije**)	**Kućno lečenje i izolacija** uz kontrole i nadzor **Kovid ambulanti**	**Vitaminska th:** alfakalcidol tbl. (**Alpha D_3**) 1x2 mcg, **vitamin C** 1x1g (ukoliko nema bubrežnih smetnji)<br>**** Ne davati antibiotike rutinski! Zbog rizika ozbiljnih neželjenih dejstava i rastuće rezistencije na antimikrobne lekove.***<br>**Antivirusna th.** (**započeti najkasnije do 5. dana** od početka tegoba): **Favipiravir** tbl. 1600 mg na 12 h, prvi dan, zatim 600 mg na 12h još 4 dana (lek se primenjuje kod obolelih koji imaju simptome)<br>**Simptomatska th.**
OBLIK 2<br>1. **Pozitivan nazofaringealni bris** (PCR na SARS-KoV-2, Ag test)<br>2. **Blaga klinička slika**<br>3. **Bolesnici bez komorbiditeta** i sa blagim oblikom infekcije (hospitalizovani pacijenti sa sO_2 > 94% **i sa Rtg znacima pneumonije sa ili bez znakova hipoksije pri prijemu**)	1. **Kovid centri:** odelj. opšteg tipa<br>2. **Opšte bolnice:** odelj. izolacije<br>Mere pojačanog nadzora<br>Više puta dnevno praćenje temperature, sO_2 i fr. disanja	**Antikoagulantna th:** Niskomolekulrni heparin<br>**Antivirusna th.** (**započeti najkasnije do 5. dana** od početka tegoba): **Favipiravir** tbl. 1600 mg na 12 h, prvi dan, zatim 600 mg na 12h još 4 dana<br>**Kortikosteroidna th.**<br>**Vitaminska th:** alfakalcidol tbl. (**Alpha D_3**) 1x2 mcg, **vitamin C** 1x1g<br>**Plazma rekonvalescenata** (unutar 2 nedelje od početka tegoba i nakon konsultacije sa infektologom i transfuziologom, prema skoru)<br>*Primena antibiotika samo kod verovatne ili dokazane bakterijske inf.*
OBLIK 3<br>1. **Pozitivan nazofaringealni bris** (PCR na SARS-KoV-2, Ag test)<br>2. **Umereno teška klinička slika**<br>3. **Teška hipoksija** sa potrebom za oksigenu Th (sO_2 <90%), febrilnost, multiple opacifikacije na Rtg-u pluća (ili specifične promene na plućima viđene na CT-u)<br>4. **Citokinska oluja** (pogoršanje opšteg stanja uz ↑CRP, ↑fibrinogen, ↑D-dimer, ↑IL-6) (bar jednog parametrta)	1. **Kovid centri:** odelj. poluintenzivne nege<br>2. **Opšte bolnice:** odelj. izolacije sa **stalnim** nadzorom (≤1h) intenziviste, anesteziologa, infektologa ili interniste<br>3. **U slučaju daljeg pogoršanja: transport u Kovid intenzivne nege**	**Antikoagulantna th:** Niskomolekulrni heparin<br>**Vitaminska th:** alfakalcidol tbl. (**Alpha D_3**) 1x2 mcg, **vitamin C** 1x1g<br>**Oksigenoterapija:** nazalni kateter ili maska (O_2 10-15 L/min)<br>**Antivirusna th.** (**ako je prošlo manje od 5. dana** od početka tegoba) **Favipiravir** (kao gore) ili **Remdesivir** amp. 200 mg i.v. prvi dan, zatim 100 mg i.v./dan još 4 dana<br>**Kortikosteroidna th.** i/ili **tocilizumab** 8 mg/kg i.v. po dozi. Daju se 2 doze (max. 800 mg/dozi)<br>**Antibiotska th:** prema vodiču za racionalnu primenu antibiotika,<br>**Plazma rekonvalescenata** (unutar 2 nedelje od početka tegoba i nakon konsultacije sa infektologom i transfuziologom, prema skoru)
OBLIK 4 i 5<br>1. **Pozitivan nazofaringealni bris**<br>2. **Veoma teška/teška klinička slika**<br>3. **Početak ili razvoj ARDS-a**<br>4. **Citokinska oluja** (pogoršanje opšteg stanja uz ↑CRP, ↑fibrinogen, ↑D-dimer, ↑IL-6)	**Kovid centri:** intenzivna nega	Primeniti **sve dostupne** mere/kombinacije intenzivnog lečenja<br>1. Što duže **održavanje na ↑ protoku O_2, MV** u slučaju pogoršanja<br>2. **Kortikosteroidna th:** Metilprednizolon 1-2 mg/kg, 3-5 dana, a zatim postupno redukovanje doze (dozu prilagoditi telesnoj masi da bi se smanjio rizik krvarenja i drugih neželjenih efekata)<br>3. **Tocilizumab:** 8 mg/kg i.v. po dozi - 2 doze (max. 800 mg/dozi)<br>4. **Imunoglobulni:** 10-20 g/dan Ig tokom 3-5 dana

Supplementary Table S4 STROBE Statement

Checklist of items that should be included in reports of ***cohort studies***

	Item No	Recommendation	Page No
**Title and abstract**	1	(*a*) Indicate the study's design with a commonly used term in the title or the abstract	1,2
		(*b*) Provide in the abstract an informative and balanced summary of what was done and what was found	1
**Introduction**			
Background/rationale	2	Explain the scientific background and rationale for the investigation being reported	1,2
Objectives	3	State specific objectives, including any prespecified hypotheses	2
**Methods**			
Study design	4	Present key elements of study design early in the paper	2
Setting	5	Describe the setting, locations, and relevant dates, including periods of recruitment, exposure, follow-up, and data collection	2, 12, 13
Participants	6	(*a*) Give the eligibility criteria, and the sources and methods of selection of participants. Describe methods of follow-up	12, 13
		(*b*) For matched studies, give matching criteria and number of exposed and unexposed	N/A
Variables	7	Clearly define all outcomes, exposures, predictors, potential confounders, and effect modifiers. Give diagnostic criteria, if applicable	12, 13
Data sources/ measurement	8*	For each variable of interest, give sources of data and details of methods of assessment (measurement). Describe comparability of assessment methods if there is more than one group	*12, 13*
Bias	9	Describe any efforts to address potential sources of bias	12, 13
Study size	10	Explain how the study size was arrived at	N/A
Quantitative variables	11	Explain how quantitative variables were handled in the analyses. If applicable, describe which groupings were chosen and why	13
Statistical methods	12	(*a*) Describe all statistical methods, including those used to control for confounding	13
		(*b*) Describe any methods used to examine subgroups and interactions	13
		(*c*) Explain how missing data were addressed	12

		(*d*) If applicable, explain how loss to follow-up was addressed	N/A
		(*e*) Describe any sensitivity analyses	N/A
**Results**			
Participants	13*	(a) Report numbers of individuals at each stage of study—eg numbers potentially eligible, examined for eligibility, confirmed eligible, included in the study, completing follow-up, and analysed	2
		(b) Give reasons for non-participation at each stage	2
		(c) Consider use of a flow diagram	N/A
Descriptive data	14*	(a) Give characteristics of study participants (eg demographic, clinical, social) and information on exposures and potential confounders	2, 3
		(b) Indicate number of participants with missing data for each variable of interest	2,3
		(c) Summarise follow-up time (eg, average and total amount)	2, 3
Outcome data	15*	Report numbers of outcome events or summary measures over time	2-9
Main results	16	(*a*) Give unadjusted estimates and, if applicable, confounder-adjusted estimates and their precision (eg, 95% confidence interval). Make clear which confounders were adjusted for and why they were included	N/A
		(*b*) Report category boundaries when continuous variables were categorized	3-9
		(*c*) If relevant, consider translating estimates of relative risk into absolute risk for a meaningful time period	N/A
Other analyses	17	Report other analyses done—eg analyses of subgroups and interactions, and sensitivity analyses	5-9
**Discussion**			
Key results	18	Summarise key results with reference to study objectives	10, 11
Limitations	19	Discuss limitations of the study, taking into account sources of potential bias or imprecision. Discuss both direction and magnitude of any potential bias	11, 12
Interpretation	20	Give a cautious overall interpretation of results considering objectives, limitations, multiplicity of analyses, results from similar studies, and other relevant evidence	10, 11, 12
Generalisability	21	Discuss the generalisability (external validity) of the study results	11, 12
**Other information**			

Funding	22	Give the source of funding and the role of the funders for the present study and, if applicable, for the original study on which the present article is based	14
